# Aortic versus non-aortic cryopreserved allograft and donor-specific outcome: a retrospective observational study

**DOI:** 10.3389/fsurg.2026.1957142

**Published:** 2026-10-09

**Authors:** Matthias Buerger, Anna Schneider, Franz Bartholomé, Philipp Pauli, Alexandra Bonietzki, Thorben Michaelis, Melanie Rusch, Grischa Hoffmann, Rene Rusch, Sebastian Kapahnke

**Affiliations:** 1 Clinic for Vascular and Endovascular Surgery, University Hospital of Schleswig-Holstein, Kiel, Germany; 2 Charité – Universitätsmedizin Berlin, Institute of Medical Sociology and Rehabilitation Science, Berlin, Germany; 3 Clinic for Angiology, University Hospital of Schleswig-Holstein, Kiel, Germany; 4 Clinic for Vascular Surgery, Medical School and University Medical Centre OWL, Bielefeld, North Rhine-Westphalia, Germany

**Keywords:** allograft, homograft, open aortic repair, peripheral vascular surgery, vascular graft infection

## Abstract

**Introduction:**

Cryopreserved arterial allografts are an established conduit for vascular reconstruction in patients with vascular graft infection (VGI). Although outcomes after aortic and peripheral implantation have been reported separately, comparative data including donor- and graft-specific characteristics are limited. This study compared clinical, procedural, and donor-related factors between aortic and non-aortic allograft implantation and evaluated their association with postoperative outcomes.

**Materials and methods:**

This retrospective single-center study included all consecutive patients undergoing arterial allograft implantation between January 2023 and December 2025. Patients were stratified into aortic position (AP; *n* = 22) and non-aortic position (NAP; *n* = 29). Clinical, procedural, microbiological, donor-, and graft-specific variables were analyzed. Primary endpoints were in-hospital and 30-day mortality. Secondary endpoints included overall survival, primary graft patency, limb salvage and reintervention.

**Results:**

Fifty-one patients received 112 cryopreserved arterial allografts in multisegment reconstructions. VGI was present in 76.5% of patients. Compared with NAP, AP reconstruction required longer operative time (343 vs. 262 min, *p* = .002) and greater blood loss (1,125 vs. 550 mL, *p* = .001). In-hospital/30-day mortality was higher after AP reconstruction (22.7% vs. 3.5%, *p* = .073), whereas primary graft patency and reintervention rates were comparable. Kaplan–Meier analysis demonstrated significantly reduced survival after aortic reconstruction (log-rank *p* = .011). Donor age, sex, graft storage duration, and transport characteristics were similar between groups. Non-aortic allografts showed significantly better histological quality (*p* = .012).

**Discussion:**

Aortic allograft reconstruction was associated with greater procedural complexity and reduced overall survival, likely reflecting the severity of underlying disease and extent of surgery. Histological graft quality differed significantly between implantation sites and may represent an underrecognized determinant of allograft outcomes.

**Conclusion:**

Cryopreserved arterial allografts provide effective vascular reconstruction in both aortic and non-aortic positions, with favorable graft patency. Inferior survival after aortic reconstruction occurred despite largely comparable donor characteristics, aside from a higher histological quality grade in NAP. This finding is hypothesis-generating and warrants further evaluation. Prospective multicenter studies should determine whether histological graft assessment improves allograft selection and predicts long-term outcomes.

## Introduction

Vascular graft infections (VGI) represent a rare yet devastating complication in septic vascular surgery. The incidence ranges from 1% to 6%, depending on the anatomical location (1, 2). In addition to antimicrobial therapy, surgical debridement with explantation of the infected material constitutes the cornerstone of successful treatment. Current concepts in septic vascular surgery encompass both *in-situ* and extra-anatomic reconstructions to preserve organ and limb perfusion. Available graft materials include synthetic prostheses (optionally rifampicin- or silver-coated), bovine materials, allogenic materials (cryopreserved allografts), and autologous materials. Autologous grafts demonstrate the lowest rates for reinfection. However, they are not universally available (2). Cryopreserved allografts offer favorable outcomes regarding infection clearance and survival but are limited by availability constraints and the risk of long-term degeneration (3). Bovine pericardial grafts have emerged as a promising alternative with good availability and comparable outcomes (4). Contrary to previously held beliefs, synthetic materials represent an acceptable alternative when the aforementioned graft options are unavailable or technically not feasible (5).

Mortality rates can be exceedingly high, ranging from 30% for peripheral infections to 56% for aortic infections (6). Beyond the presence of significant comorbidities and patient age, the causative pathogen spectrum as well as characteristics of the index procedure (emergency surgery, operation time, postoperative complications, and reoperations) constitute relevant risk factors for high morbidity and mortality following VGI (2, 7, 8). Additionally, the anatomical location of the infection (aortic vs. peripheral) represents an important prognostic factor (6).

Regarding long-term treatment success with allografts in septic surgery, donor-specific factors such as age may also play a significant role (9). Whether donor-specific characteristics such as age or sex, as well as processing-related and graft-specific characteristics such as storage duration, diameter, and histological quality grade differ between aortic and peripheral allograft sites has not been investigated to date. To obtain a comprehensive overview of outcomes following VGI treated with allograft implantation in aortic vs. non-aortic positions, the aim of this retrospective observational study was to compare clinical, procedural and donor-specific parameters with regard to survival and postoperative complications.

## Material and methods

The current study was approved by the local ethics review committee of the University Hospital Schleswig-Holstein, Kiel, Germany (protocol identification number: D 407/26). All procedures, data proceedings and analyses were performed in accordance with the Helsinki Declaration of 2013. The manuscript was prepared using the Strengthening the Reporting of Observational Studies in Epidemiology (STROBE) Statement guidelines (10).

### Sample collection and study design

The study was conducted at the Clinic for Vascular and Endovascular Surgery, University Hospital Schleswig-Holstein, Kiel, Germany. This single-center retrospective observational study included all patients who underwent implantation of an arterial allograft between January 2023 and December 2025. Clinical data were retrieved from the hospital information system, while donor-specific data were obtained from standard implantation records provided by the German Society for Tissue Transplantation (Deutsche Gesellschaft für Gewebetransplantation, DGFG). Eligible participants included all patients who received an arterial allograft due to VGI, inflammatory vascular disease or systemic inflammation and absence of suitable autologous conduit. Patients undergoing vascular reconstruction as part of oncologic tumor resection were excluded. Participants were identified through a procedural code search within the hospital information system. Follow-up was based on the last documented patient contact or hospital visit recorded in the institutional database, without additional follow-up attempts or external contact to patients or primary care providers. Postoperative surveillance followed institutional standard practice, consisting of duplex ultrasonography at discharge, three and six months and annually thereafter supplemented by contrast-enhanced CT angiography in cases of clinical suspicion of complications. To minimize bias, data collection was performed by M.B. and independently verified by S.K. for accuracy and completeness. The study size was determined by a retrospective inclusion of all eligible patients who underwent arterial allograft implantation during the predefined study period, resulting in a total sample size of *N* = 51 cases.

The primary outcomes of the study were in-hospital and 30-day mortality. Secondary outcomes included long-term survival and the need for reintervention due to graft failure defined as loss of primary graft patency, graft-related amputations or graft-reinfection [defined according to Management of Aortic Graft Infection Collaboration (MAGIC) criteria]. The primary exposure was implantation site, categorized as aortic position (AP; *n* = 22) vs. non-aortic position (NAP; *n* = 29). Predictive variables comprised donor- and graft-specific characteristics (e.g., donor age, sex, graft transport temperature, storage duration, graft diameter, histological quality and evidence of microbiological contamination in transport medium) and clinical parameters (e.g., patient age, gender, comorbidities, indication, previous vascular procedures, microbiological specimens, inflammatory markers, duration of intensive care unit- and hospital stay, complications) as well as procedural parameters (e.g., operative time and blood loss). Each indication for allograft implantation was determined on an interdisciplinary basis (angiology, interventional radiology and vascular surgery). The diagnosis of VGI was made using the MAGIC criteria (11) in accordance with the Clinical Practice Guidelines on the Management of Vascular Graft and Endograft Infections (1). Inflammatory vascular diseases were diagnosed in accordance with rheumatological recommendations based on a combination of clinical symptoms, laboratory findings (e.g., elevated inflammatory markers), imaging, and biopsy where appropriate. Systemic inflammation and lack of suitable autologous conduit options were evaluated after combining clinical, laboratory, and sonographic diagnostics.

### Allograft preparation prior to implantation

The donor vessel was collected in accordance with the DGFG's standard protocol (12). A standardized quality assessment of the allografts was performed according to a protocol established by the DGFG. Based on the American Heart Association (AHA) scoring system, vascular grafts were classified into five quality grades. Grade 4 represents the highest quality, whereas Grade 0 indicates the lowest quality. Only allografts achieving a quality grade of 3 (corresponding to AHA classification types 3–5) or 4 (corresponding to AHA classification types 1 and 2) were eligible for clinical use. Vascular grafts assigned a lower quality grade (≤ grade 2, corresponding to AHA classification type 6) were discarded in accordance with the standard operating procedure VA59-HKK (Rejection and Handling of Discarded Tissue). A detailed description of the histological criteria distinguishing each AHA-based grade is provided in Supplementary Table S1. Prior to implantation, allografts were repeatedly thawed in prewarmed water (37 °C) following the general recommendations provided by the DGFG. Subsequently, the allografts were rinsed repeatedly with heparinized saline at room temperature and sampled for bacteriology culture.

### Statistical data analysis

Data were collected in Microsoft Excel (Microsoft, Redmond, Washington, USA) and analyzed using GraphPad Prism 11 (GraphPad Software, San Diego, California, USA). Binary and categorical variables were compared using chi-square or Fisher's exact tests. Continuous variables were tested for normality using the D'Agostino-Pearson test and analyzed with unpaired *t*-tests (normal distribution) or Mann–Whitney *U*-tests (non-normal distribution). Normally distributed data are presented with mean ± standard deviations (SD), while non-normally distributed data are presented with median with interquartile ranges (IQR). Survival analysis was performed using Kaplan–Meier curves with log-rank test for group comparisons. Statistical significance was set at *p* < .05.

## Results

### General characteristics and indications

The median age of the 51 patients was 65 ± 13 years. Fifteen (29%) of the patients were women. The general characteristics of the AP and NAP groups are shown in Table 1. No significant differences were found in terms of age or gender. Apart from a higher incidence (AP: 0.00% vs. NAP: 20.69%; *p* = .031) of minor tissue loss, corresponding to Peripheral Artery Disease Rutherford Grade 5, there were no significant differences in comorbidities among both groups.

**Table 1:** Patient demographics, indications and preoperative characteristics.

**Parameter**	**Aortic position (*n* = 22)**	**Non-aortic position (*n* = 29)**	***p*-value**
Sex: Women	7 (31.82%)	8 (27.59%)	.766
Age (years)	65 ± 11	65 ± 15	.854
BMI (kg/m^2^)	25.47 ± 3.89	26.27 ± 5.25	.537
Arterial Hypertension	14 (63.64%)	25 (86.21%)	.095
Diabetes Mellitus	2 (9.09%)	9 (31.03%)	.088
Chronic Obstructive Pulmonary Disease	2 (9.09%)	3 (10.34%)	>.999
Coronary Heart Disease	11 (50.00%)	13 (44.83%)	.782
Atrial Fibrillation	4 (18.18%)	4 (13.79%)	.713
Hypercholesterolemia	13 (59.09%)	21 (72.41%)	.377
Congestive Heart Failure	3 (13.64%)	2 (6.90%)	.641
Myocardial Infarction < 6 months	1 (4.55%)	1 (3.45%)	>.999
Peripheral Artery Disease	3 (13.64%)	12 (41.38%)	.061
Rutherford Grade 1	1 (4.55%)	3 (10.34%)	.625
Rutherford Grade 2	0 (0.00%)	1 (3.45%)	>.999
Rutherford Grade 3	2 (9.09%)	2 (6.90%)	>.999
Rutherford Grade 4	0 (0.00%)	0 (0.00%)	>.999
Rutherford Grade 5	0 (0.00%)	6 (20.69%)	**.031**
Rutherford Grade 6	0 (0.00%)	0 (0.00%)	>.999
Smoking	11 (50.00%)	22 (75.86%)	.078
Renal Insufficiency			
Stadium 1	7 (31.82%)	10 (34.48%)	>.999
Stadium 2	8 (36.36%)	8 (27.59%)	.554
Stadium 3a	3 (13.64%)	6 (20.69%)	.714
Stadium 3b	0 (0.00%)	4 (13.79%)	.124
Stadium 4	3 (13.64%)	1 (3.45%)	.303
Stadium 5	1 (4.55%)	0 (0.00%)	.431
Stadium 6	0 (0.00%)	0 (0.00%)	>.999
ASA Physical Status Classification System			
ASA 1	0 (0.00%)	1 (3.45%)	>.999
ASA 2	2 (9.09%)	4 (13.79%)	.688
ASA 3	15 (68.18%)	21 (72.41%)	.766
ASA 4	5 (22.73%)	3 (10.34%)	.268
ASA 5	0 (0.00%)	0 (0.00%)	>.999
ASA 6	0 (0.00%)	0 (0.00%)	>.999
Preoperative C-reactive protein (mg/dL)	21.55 [10.20–56.15]	44.50 [6.41–83.25]	.717
Preoperative Leukocytes (10^9^/L)	9.49 ± 3.50	9.58 ± 2.90	.925
MAGIC-Criteria			
Suspected VGI	6 (27.27%)	7 (24.14%)	>.999
Diagnosed VGI	9 (40.91%)	17 (58.62%)	.264
Previous Vascular Procedure	15 (68.18%)	25 (86.21%)	.173
Number of Previous Vascular Procedures	1.00 [0.00–3.75]	4.00 [1.00–6.50]	**.047**
Open Procedure	0.00 [0.00–1.00]	2.00 [1.00–4.00]	**.001**
Endovascular Procedure	0.00 [0.00–1.25]	1.00 [0.00–2.50]	.502
Duration last Vascular Procedure to Clinical Presentation (days)	98.00 [37.50–2,100.00]	39.00 [18.50–341.00]	.155
Early vs. Late VGI			
Early VGI	7 (31.82%)	15 (51.72%)	.253
Late VGI	8 (36.36%)	9 (31.03%)	.769
Material VGI			
Dacron Intergard Synergy	6 (27.27%)	12 (41.38%)	.380
Dacron	2 (9.09%)	2 (6.90%)	>.999
Bovine	1 (4.55%)	5 (17.24%)	.218
Nitinol	1 (4.55%)	1 (3.45%)	>.999
PTFE	0 (0.00%)	2 (6.90%)	.500
PTFE and Nitinol	5 (22.73%)	2 (6.90%)	.216
Silver	0 (0.00%)	1 (3.45%)	>.999
WHD at Clinical Presentation	2 (9.09%)	13 (44.83%)	**.006**
WHD at Clinical Presentation Samson-Classification			
Grade 1	0 (0.00%)	0 (0.00%)	>.999
Grade 2	0 (0.00%)	1 (3.45%)	>.999
Grade 3	0 (0.00%)	7 (24.14%)	**.015**
Grade 4	2 (9.09%)	4 (13.79%)	.688
Grade 5	0 (0.00%)	1 (3.45%)	>.999
Microbiological Contamination Wound at Clinical Presentation	2 (9.09%)	11 (37.93%)	**.025**
Blood Culture Positive at Clinical Presentation	2 (9.09%)	3 (10.34%)	>.999
Microbiological Contamination Wound Intraoperative	4 (18.18%)	15 (51.72%)	**.020**
Microbiological Contamination Graft Intraoperative	3 (13.64%)	5 (17.24%)	>.999
Antibiotic Treatment preoperative	14 (63.64%)	24 (82.76%)	.194
Antibiotic Treatment postoperative	20 (90.91%)	27 (93.10%)	>.999
Duration of Antibiotic Treatment (days)	10 [7.00–20.00]	27.00 [12.00–42.00]	**.006**

BMI, Body Mass Index; ASA, American Society of Anesthesiologists; ICU, Intensive Care Unit, MAGIC, Management of Aortic Graft Infection Collaboration, VGI, Vascular Graft Infection; WHD, Wound Healing Disorder. Continuous variables are presented as Mean ± Standard Deviation (normally distributed) or median and interquartile range (non-normal distribution) and categoric dichotomous variables as Counts and Percentages, unless otherwise stated. Significant *p*-values are shown in bold.

According to the MAGIC criteria, a total of 39 (76.47%) patients showed signs of either suspected or diagnosed VGI. Neither VGI was suspected nor diagnosed in the remaining twelve (23.53%) patients. Four (7.84%) patients had inflammatory vascular disease. Indications included one case of Takayasu's arteritis (1.96%), two cases of Giant Cell Arteritis (3.92%) and one case of Behçet's disease (1.96%). Two (3.92%) patients met either minor or major criteria of the MAGIC classification. One patient (1.96%) presented with perigraft fluid only while another patient (1.96%) had positive blood cultures and a pseudoaneurysmal complication. Four (7.84%) patients did not have any vascular graft material implanted. One patient (1.96%) presented with highly elevated inflammatory markers and positive blood cultures in gangrene with no suitable autologous conduit. Another patient (1.96%) had positive blood cultures and a large aneurysm of the proximal superior mesenteric artery. Due to multiple intolerances, an infrarenal aortic aneurysm was replaced with an allograft at one patient's (1.96%) explicit request. Another patient (1.96%) presented with a ruptured aortic aneurysm accompanied by adjacent spondylodiscitis with psoas abscess, leading to the decision to perform emergency allograft replacement. One further patient (1.96%) experienced recurrent early occlusions with both endovascular stent grafts and open surgical prosthetic materials. Due to an endocarditis associated with contamination of a cardiovascular bioprosthesis in the ascending aorta, an allograft was implanted to replace the abdominal aortic aneurysm in one (1.96%) patient.

Overall, there were no differences in the frequency of emergent/urgent procedures between the two groups (AP: 22.73% vs. NAP: 20.69%; *p* > .999). The non-aortic position patients had undergone more previous vascular treatments [AP: 1.00 [0.00–3.75] vs. 4.00 [1.00–6.50]; *p* = .047], particularly open surgery procedures [AP: 0.00 [0.00–1.00] vs. 2.00 [1.00–4.00]; *p* = .001].

### Wound characteristics and microbiological contamination

Wound healing disorder (WHD) in general (AP: 9.09% vs. NAP: 44.83%; *p* = .006) and deep VGI classified as Samson grade 3 (AP: 0.00% vs. NAP: 24.12%; *p* = .015) were observed more often in the non-aortic than the aortic position allograft group (Table 1). Patients in the non-aortic position group presented more frequently with a microbiological contamination of the WHD at presentation (AP: 9.09% vs. NAP: 37.93%; *p* = .025) and in intraoperative specimen analyses (AP: 18.18% vs. NAP: 51.72%; *p* = .020).

The preoperative microbiological spectrum differed markedly between groups (Supplementary Material Table S2). In the aortic position group, preoperative and intraoperative specimens revealed four gram-positive (18.18%) and five gram-negative (22.73%) pathogen detections, with no evidence of polymicrobial colonization. In the non-aortic position group, isolates were predominantly gram-positive with 16 cases (55.17%), followed by nine gram-negative pathogens (31.03%), while polymicrobial colonization was identified in five cases (17.24%). Blood cultures were positive for Staphylococcus aureus in all three bacteremic patients. Antimicrobial treatment showed considerable variability and is described in Supplementary Material Table S3 in detail.

### Procedural characteristics

The average operative time for all reconstructive procedures was 297 ± 91 min. The average intraoperative blood loss for all procedures was 1,042 ± 882 mL (Table 2). In the aortic group, aortobiiliac bypass was the most common performed procedure (*n* = 8, 36.36%), followed by tubular grafts (*n* = 5, 22.73%) combined with aortomesenteric bypass in one patient (*n* = 1, 4.55%), aortofemoral bypass (*n* = 3, 13.64%), aortoiliac bypass (*n* = 3, 13.64%) and aortobifemoral bypass (*n* = 2, 9.09%, see Figure 1). In the non-aortic group, iliacofemoral interponates were most common (*n* = 9, 31.03%), followed by iliacofemoral bypass (*n* = 8, 27.59%) and popliteal interpositions (*n* = 5, 17.24%). Less frequent procedures included femoral interpositions (*n* = 3, 10.34%), femoropopliteal bypass (*n* = 2, 6.90%), and single cases of femoral patch reconstruction (3.45%) and aortomesenteric interposition (3.45%).

**Table 2:** Procedural data.

**Parameter**	**Aortic position (*n* = 22)**	**Non-aortic position (*n* = 29)**	***p*-value**
Operative Time (min)	342.82 ± 93.90	261.66 ± 72.84	**.** **002**
Blood Loss during Surgery (mL)	1,125.00 [690.00–2,350.00]	550.00 [375.00–825.00]	**.001**
Number Red Blood Cell Transfusions (unit)	4.00 [2.00–9.00]	1.00 [0.00–3.00]	**.001**

Continuous variables are presented as Mean ± Standard Deviation (normally distributed) or median and interquartile range (non-normal distribution), unless otherwise stated. Significant *p*-values are shown in bold.

**Figure 1:**
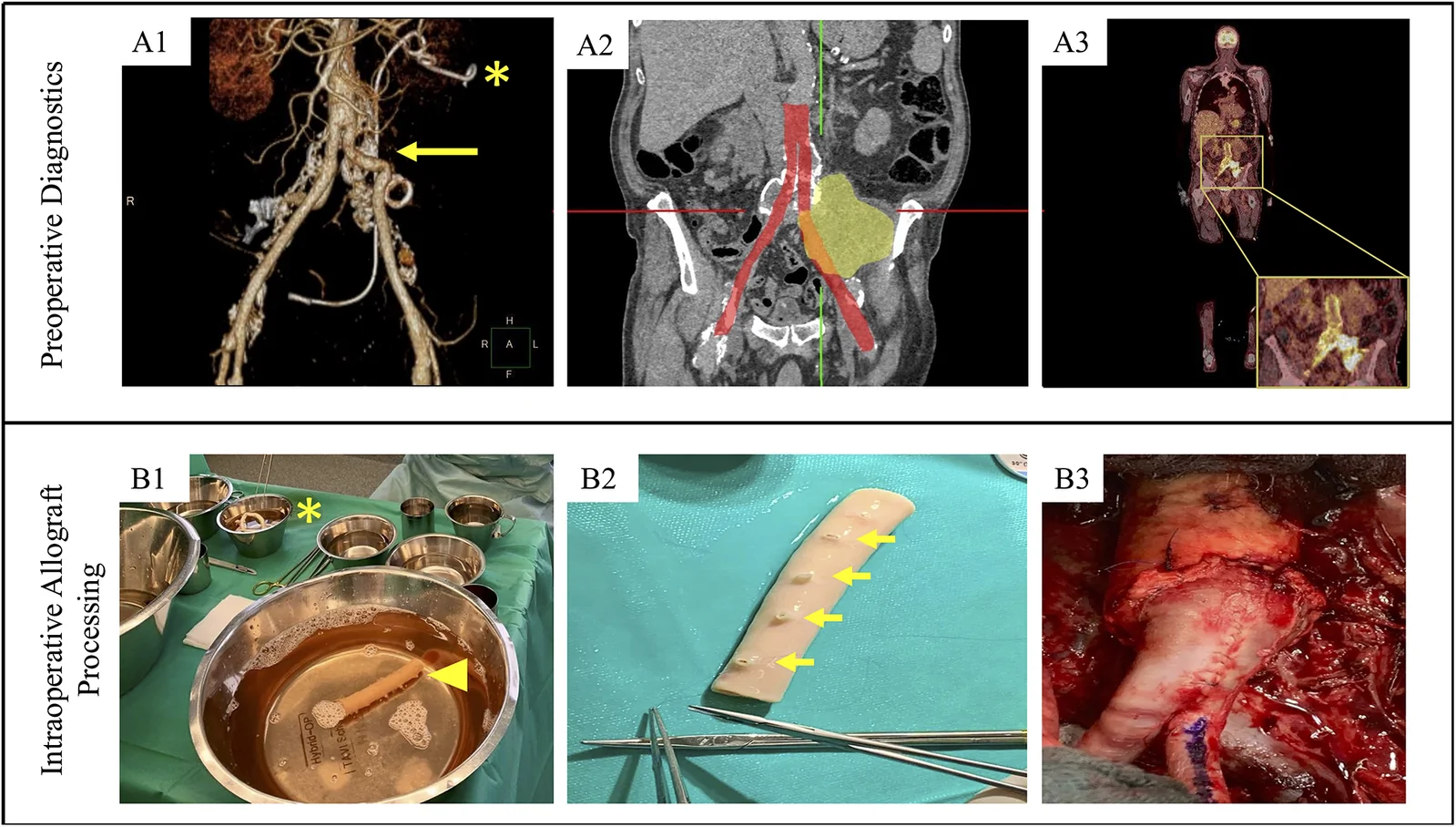
Case presentation of a 72-year-old man with a history of open repair of an aortobiiliac aneurysm by implantation of an aortobifemoral Y-graft (**A**1) at an outside institution. The index procedure was complicated by iatrogenic injury to the left ureter, resulting in persistent periprosthetic urinary leakage managed with a left double-J ureteral (A1, arrow) stent and percutaneous nephrostomy (A1, asterisk). One year later, the patient presented with a periprosthetic fluid formation (A2, aortobifemoral bypass red color, periprosthetic fluid formation yellow color) and increased 18F-fluorodeoxyglucose uptake on PET/CT (A3), consistent with vascular graft infection, accompanied by a pre-septic clinical condition and positive blood cultures. Radical debridement with complete graft explantation, concomitant left nephrectomy, and *in situ* reconstruction using an aortobifemoral arterial allograft was performed. One thoracic (**B**1, arrowhead) and four peripheral (B1, asterisk) arterial allografts were prepared, *ex vivo* suture closure of the thoracic aortic side branches (B2, arrows), and used to construct a custom-made infrarenal aortobifemoral allograft with a neo-bifurcation created from the peripheral allografts (B3).

Compared to non-aortic position allografts, aortic procedures showed significantly longer operative times (AP: 342.82 ± 93.90 vs. NAP: 261.66 ± 72.84 min; *p* = .002), greater blood loss [AP: 1,125.00 [690.00–2,350.00] vs. NAP: 550.00 [375.00–825.00] mL; *p* = .001], and more Red Blood Cell transfusions [AP: 4.00 [2.00–9.00] vs. NAP: 1.00 [0.00–3.00] units; *p* = .001].

### Hospital course during index admission

Aortic vs. non-aortic position allografts showed no difference in duration of stay at the intensive care unit [Table 3; AP: 2.00 [1.00–6.50] vs. NAP: 4.00 [1.00–6.00] days; *p* = .843]. Clavien-Dindo classification grades differed significantly with Grade 2 being more frequent in aortic (AP: 59.09% vs. NAP: 6.90%; *p* = .001), Grade 3b in non-aortic (AP: 4.55% vs. NAP: 55.17%; *p* = .001) and Grade 5 numerically higher in the aortic group (AP: 22.73% vs. NAP: 3.45%; *p* = .073). Aortic cases had more paralytic ileus (AP: 36.36% vs. NAP: 0.00%; *p* = .001) and AKIN 3 acute kidney injury (AP: 18.18% vs. NAP: 0.00%; *p* = .029), while non-aortic cases showed longer hospital stays [AP: 12.00 [8.75–20.25] vs. NAP: 24.00 [13.50–42.50] days; *p* = .004] and a higher total number of surgeries [AP: 0.00 [0.00–2.00] vs. NAP: 1.00 [0.00–5.00]; *p* = .008].

**Table 3:** Hospital course and complications during index admission.

**Parameter**	**Aortic position (*n* = 22)**	**Non-aortic position (*n* = 29)**	***p*-value**
ICU Length of Stay (days)	2.00 [1.00–6.50]	4.00 [1.00–6.00]	.843
Clavien-Dindo Classification			
Grade 1	1 (4.55%)	6 (20.69%)	.124
Grade 2	13 (59.09%)	2 (6.90%)	**.001**
Grade 3a	0 (0.00%)	0 (0.00%)	>.999
Grade 3b	1 (4.55%)	16 (55.17%)	**.001**
Grade 4a	1 (4.55%)	3 (10.34%)	.625
Grade 4b	1 (4.55%)	1 (3.45%)	>.999
Grade 5	5 (22.73%)	1 (3.45%)	.073
Peripheral Ischemia	2 (9.09%)	5 (17.24%)	.684
Amputation	0 (0.00%)	2 (6.90%)	.500
Minor Amputation	0 (0.00%)	1 (3.45%)	>.999
Major Amputation	0 (0.00%)	1 (3.45%)	>.999
Compartment Syndrome	1 (4.55%)	1 (3.45%)	>.999
Bleeding	4 (18.18%)	4 (13.79%)	.713
Paralytic Ileus	8 (36.36%)	0 (0.00%)	**.001**
Nonocclusive Mesenteric Ischemia	3 (13.64%)	0 (0.00%)	.074
Myocardial Dysfunction	3 (13.64%)	1 (3.45%)	.303
Pneumonia	6 (27.27%)	3 (10.34%)	.150
Respiratory Failure	6 (27.27%)	2 (6.90%)	.063
WHD Postoperative	3 (13.64%)	4 (13.79%)	>.999
WHD Postoperative Samson-Classification			
Grade 1	0 (0.00%)	1 (3.45%)	>.999
Grade 2	1 (4.55%)	2 (6.90%)	>.999
Grade 3	1 (4.55%)	1 (3.45%)	>.999
Grade 4	1 (4.55%)	1 (3.45%)	>.999
Grade 5	0 (0.00%)	0 (0.00%)	>.999
Urinary Tract Infection	3 (13.64%)	4 (13.79%)	>.999
Acute Kidney Injury			
AKIN 1	1 (4.55%)	0 (0.00%)	.431
AKIN 2	0 (0.00%)	4 (13.79%)	.124
AKIN 3	4 (18.18%)	0 (0.00%)	**.029**
Total Number of Surgeries	0.00 [0.00–2.00]	1.00 [0.00–5.00]	**.008**
Hospital Stay (days)	12.00 [8.75–20.25]	24.00 [13.50–42.50]	**.004**

ICU, Intensive Care Unit; WHD, Wound Healing Disorder; AKIN,- Acute Kidney Injury Network. Continuous variables are presented as Mean ± Standard Deviation (normally distributed) or median and interquartile range (non-normal distribution) and categoric dichotomous variables as Counts and Percentages, unless otherwise stated. Significant *p*-values are shown in bold.

### Mortality and follow up

In-hospital-/30-day outcomes and follow-up were comparable between aortic and non-aortic allograft position (Table 4). While only one patient died in the non-aortic position group, with five patients in-hospital-/30-day mortality was numerically higher after aortic procedures (AP: 22.73% vs. NAP: 3.45%; OR 8.24, 95% CI 0.91–99.80; *p* = .073). In-hospital primary graft patency was similar (AP: 95.45% vs. NAP: 82.76%; RR 1.15, 95% CI 0.92–1.47; *p* = .218). Median follow-up was 193 days [(99.00–454.00)]. During follow-up, rates of re-intervention (AP: 22.73% vs. NAP: 20.69%; RR 1.10, 95% CI 0.39–2.99; *p* > .999) for indications such as graft thrombosis, pseudoaneurysm, bleeding, re-infection, wound-healing disorders, and overall primary graft patency (AP: 81.82% vs. NAP: 75.86%; RR 1.08, 95% CI 0.78–1.47; *p* = .737) did not differ significantly between aortic and non-aortic positions. The rate of death during follow-up was higher after aortic procedures (AP: 13.64% vs. NAP: 6.90%; RR 3.30, 95% CI 0.81–13.80; *p* = .216). Three patients died during follow-up in the aortic position group. Two patients died due to prolonged intestinal ischemia caused by a bypass occlusion and mechanical ileus on days 58 and 137, respectively, despite an initially uncomplicated course of treatment. Another patient died after 41 days due to bleeding caused by anastomotic leakage. Two patients in the non-aortic position group died during follow-up. One patient died 348 days after allograft-surgery from complications arising from a different surgical procedure (multiple thromboses involving the vena cava and subsequent pulmonary embolism). Another patient died 106 days after allograft implantation due to a malignant stroke.

**Table 4:** Primary and secondary outcome parameters.

**Parameter**	**Aortic position (*n* = 22)**	**Non-aortic position (*n* = 29)**	***p*-value**
In-Hospital/30d-Mortality	5 (22.73%)	1 (3.45%)	.073
Primary Graft Patency in Hospital	21 (95.45%)	24 (82.76%)	.218
Need for Re-Intervention during FU	5 (22.73%)	6 (20.69%)	>.999
Graft Thrombosis	2 (9.09%)	2 (6.90%)	>.999
Pseudoaneurysm	0 (0.00%)	1 (3.45%)	>.999
Bleeding	1 (4.55%)	0 (0.00%)	.431
Re-Infection	2 (9.09%)	1 (3.45%)	.571
WHD	1 (4.55%)	3 (10.34%)	.625
WHD FU Samson-Classification			
Grade 1	0 (0.00%)	2 (6.90%)	.500
Grade 2	0 (0.00%)	0 (0.00%)	>.999
Grade 3	1 (4.55%)	1 (3.45%)	>.999
Grade 4	0 (0.00%)	0 (0.00%)	>.999
Grade 5	0 (0.00%)	0 (0.00%)	>.999
Duration Discharge to Re-Admission (days)	51.00 [27.00–183.50]	53.00 [12.25–96.00]	.550
Number of Re-Interventions	1.00 [1.00–2.50]	2.00 [1.50–4.00]	.238
Open Surgery	4 (18.18%)	5 (17.24%)	>.999
Endovascular Intervention	1 (4.55%)	3 (10.34%)	.625
Graft Patency during FU	18 (81.82%)	22 (75.86%)	.737
Death during FU	3 (13.64%)	2 (6.90%)	.216

FU, Follow up; WHD, Wound Healing Disorder. Continuous variables are presented as Mean ± Standard Deviation (normally distributed) or median and interquartile range (non-normal distribution) and categoric dichotomous variables as Counts and Percentages, unless otherwise stated.

A Kaplan–Meier analysis (Figure 2) revealed a significant difference in overall survival between the aortic and non-aortic homograft groups (log-rank test, *p* = .011; HR 4.92, 95% CI 1.43–16.90), while no differences in time of freedom from re-intervention have been observed (log-rank test, *p* = .816).

**Figure 2:**
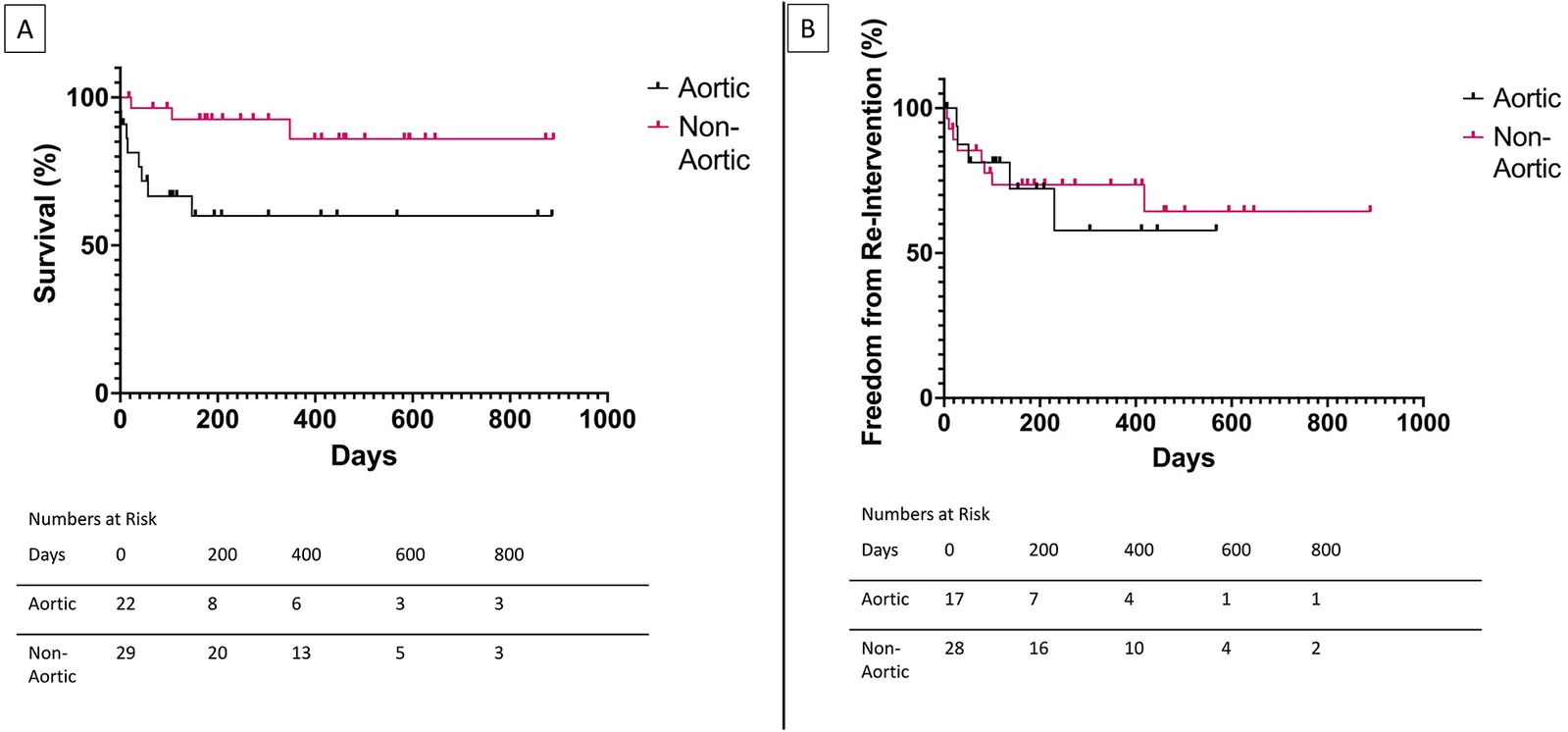
Kaplan–meier analysis for survival **(A)** and freedom from re-intervention **(B)** with corresponding tables showing numbers at risk (below).

### Donor- and allograft-specific characteristics

In total, 112 arterial allografts were implanted in 51 patients (see Table 5). The number of allografts used was significantly higher in the group with aortic allograft position [AP: 3.00 [2.00–4.00] vs. NAP: 1.00 [1.00–2.00]; *p* = .002]. Non-aortic position allografts demonstrated superior histological quality, with 48 (92.31%) graded as grade 4 (AHA classification type 1 and 2) compared to 44 (73.33%) in the aortic position group (*p* = .012). Overall microbiological contamination of the transport medium prior to cryopreservation was frequently observed in both groups, with no significant difference (AP: 70.00% vs. NAP: 65.38%, *p* = .686). Contamination was classified as monobacterial in 47 grafts (41.96%) and polybacterial (≥ two pathogens) in 29 cases (25.89%). The microbiological spectrum demonstrated considerable heterogeneity and is illustrated in Figure 3. Microbiological examinations of the allografts performed after cryopreservation immediately prior to implantation showed that all implanted allografts were sterile (100.00%).

**Table 5:** Donor-specific characteristics.

**Parameter**	**Aortic position (*n* = 60)**	**Non-aortic position (*n* = 52)**	***p*-value**
Female Donor	31 (51.67%)	21 (40.38%)	.259
Donor Age (years)	47.54 ± 11.36	43.88 ± 11.71	.198
Transport Temperature ( °C)	2.20 [1.00–2.60]	1.40 [1.00–2.35]	.223
Microbiological Contamination Transport Medium	42 (70.00%)	34 (65.38%)	.686
Duration cryopreserved Storage (days)	199.50 [69.00–503.50]	157.00 [80.00–396.00]	.763
Length (mm)	164.42 ± 48.88	192.69 ± 56.53	**.008**
Proximal Diameter (mm)	5.00 [4.00–16.75]	4.50 [4.00–5.00]	**.002**
Distal Diameter (mm)	4.00 [3.00–14.00]	4.00 [3.00–4.00]	**.014**
Histological Quality			
Grade 3 (AHA 3–5)	16 (26.67%)	4 (7.69%)	**.012**
Grade 4 (AHA 1 and 2)	44 (73.33%)	48 (92.31%)	**.012**

AHA, American Heart Association. Continuous variables are presented as Mean ± Standard Deviation (normally distributed) or median and interquartile range (non-normal distribution) and categoric dichotomous variables as Counts and Percentages, unless otherwise stated. Significant *p*-values are shown in bold.

**Figure 3:**
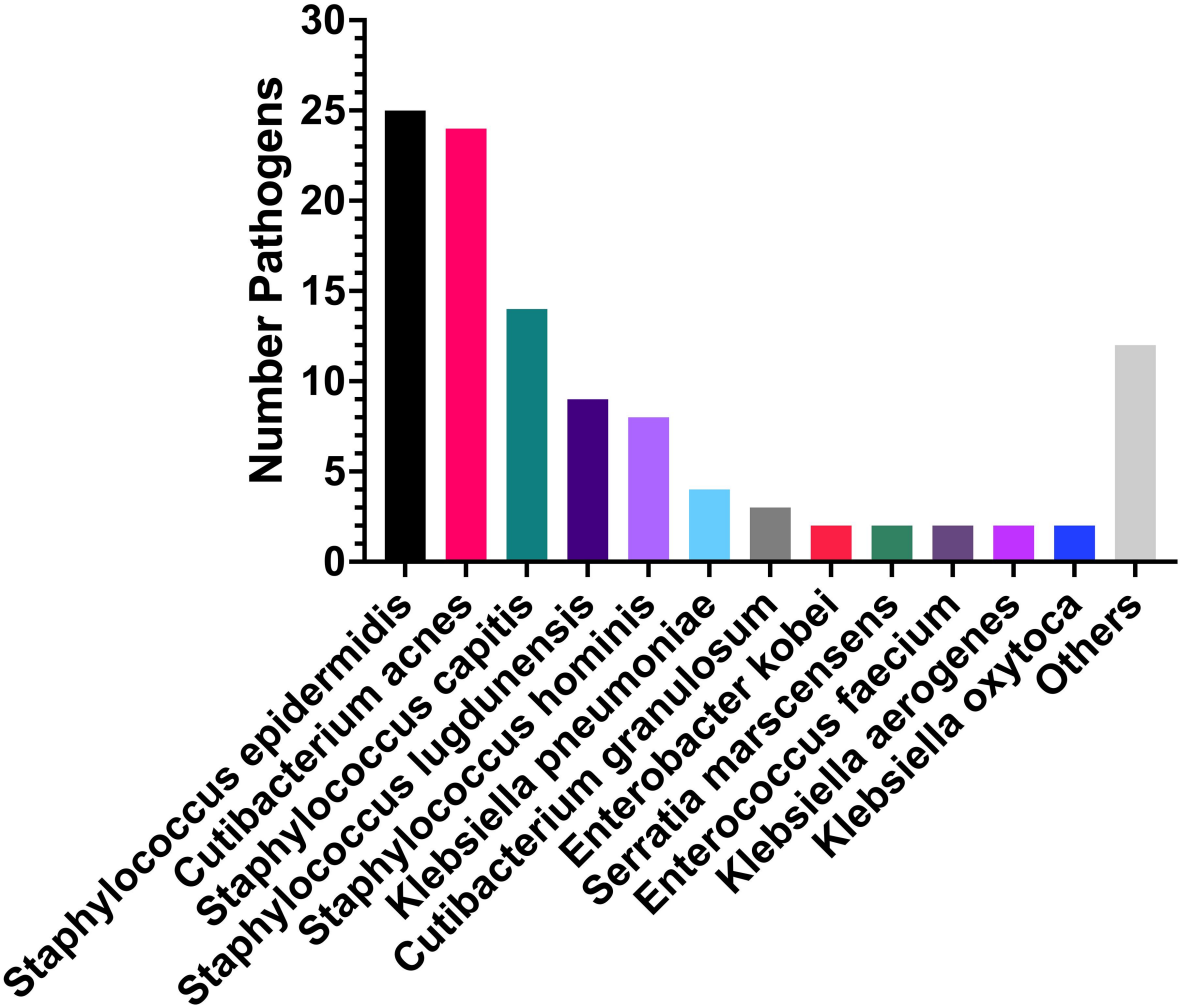
Bar chart showing the microbiological spectrum of pathogens in the transport medium of the allografts. The category “Others” includes: Corynebacterium glucuronolyticum, Enterobacter cloacae, Brevibacillus borstelensis, Chrysobacterium hominis, Staphylococcus aureus, Streptococcus parasanguinis, Serratia ureilytica, Klebsiella variicola, Staphylococcus pettenkoferi, Proteus mirabilis, Candida albicans, and Saccharomyces.

## Discussion

This study provides a detailed analysis of cryopreserved arterial allografts used for vascular reconstruction in aortic and non-aortic positions. While the vast majority of cases involved vascular graft infection, the heterogeneity of indications reflects real-world clinical practice. The principal findings include a significantly reduced overall survival rate in patients undergoing aortic position allograft reconstruction, likely reflecting greater disease severity and surgical trauma rather than an independent effect of the implantation site, as well as notable differences in donor-specific characteristics. The latter, particularly histological quality, represents a hypothesis-generating observation that warrants further investigation, in addition to the greater surgical trauma represented by longer operation time and greater blood loss.

### Factors influencing outcome in aortic allograft reconstructions

In the present study, the 30-day mortality rate was 22.73% in the aortic position group, although this estimate is based on a small subgroup sample and should be interpreted with caution. These findings are nonetheless broadly consistent with previously published data. For aortic allograft reconstruction, a systematic review and meta-analysis by Antonopoulos et al. reported a pooled 30-day mortality of 15% across 1,377 patients (13). Single-center studies have demonstrated considerable variability, with 30-day mortality rates ranging from 11% to 28% depending on patient selection and the proportion of emergency procedures (14, 15). Short- and midterm mortality rates exceed up to 56% (6). Several clinical factors have been identified as independent predictors of mortality following aortic allograft reconstruction. In a multivariate survival analysis by Touma et al., age, chronic renal disease, coronary artery disease, prosthetic graft infection as the underlying indication and emergency surgery were identified as independent predictors of postoperative mortality (14). Further patient-related predictors of adverse outcomes after cryopreserved allograft implantation include type 1 diabetes mellitus and an American Society of Anesthesiologists class 4 status (16). The baseline characteristics of our aortic position cohort were comparable to those reported in previous studies, with a mean age of 65 years aligning closely with the cohorts described by Touma et al. (14) (64 years), Couture et al. (15) (64 years), and Ben Ahmed et al. (16) (65 years). The prevalence of coronary artery disease and the proportion of ASA class 4 patients were consistent with these comparative cohorts, reflecting the high cardiovascular disease burden in this patient population. Notably, the prevalence of diabetes mellitus in our cohort (type 2 diabetes mellitus 9.09%, no type 1 diabetes mellitus) was lower than in previous studies, which may have contributed to favorable outcomes. With an emergent surgery rate of 21.15%, our study was comparable to previous studies (14, 16). Even though no operating times were reported in some comparative studies (15, 16), the procedural characteristics of our aortic position cohort reflect the inherent complexity of aortic allograft reconstruction, which plausibly contributes to the observed survival difference (14). The microbiological spectrum of VGIs in general is dominated by gram-positive cocci. Gram-negative bacteria, particularly Pseudomonas aeruginosa, predominate in intracavitary infections, whereas gram-positive organisms are more frequent in extracavitary grafts (2, 17). In our aortic position group, Pseudomonas aeruginosa was the most frequently isolated pathogen, consistent with the predominance of gram-negative organisms in aortic graft infections (2). The low rate of positive preoperative wound swabs due to absence of obvious WHD after open surgical repair or EVAR, reflects the often indolent presentation and diagnostic challenge in this setting. Beyond hybrid operating room capability (18), optimal management of these complex reconstructions likely also depends on the stability of the surgical team and nursing staff (19), dedicated intensive care infrastructure for perioperative surveillance and close interdisciplinary collaboration with infectious disease and microbiology specialists, which both may further contribute to mitigating the procedural risks described above (2).

### Factors influencing outcome in non-aortic allograft reconstructions

The 30-day mortality rate of 3.45% observed in our non-aortic position group compares favorably with previously published data on peripheral allograft implantation in the context of vascular graft infections. According to the current literature, mortality rates following peripheral allograft implantation range from approximately 6.5% to 30% within the first year, which is considerably lower than those reported for aortic allograft reconstructions (6, 20, 21). Mortality following peripheral allograft implantation for VGI is influenced by age >70 years, WHD Samson classification grades 3–5, and infections caused by Methicillin-resistent Staphylococcus aureus (MRSA) or Pseudomonas aeruginosa (2, 21). In our non-aortic position group, the mean age was 65 years, below the aforementioned critical threshold of 70 years, which may partially explain the favorable mortality rate of 3.45% in our cohort. The microbiological spectrum was dominated by Staphylococcus aureus and Staphylococcus epidermidis, with only one case of Pseudomonas aeruginosa and no MRSA detected. The favorable microbiological profile with the absence of highly virulent organisms may have contributed to the low mortality rate in this cohort. The observed mean operative time of 262 min and median blood loss of 550 mL in our cohort are consistent with the procedural complexity described for peripheral allograft reconstructions, although direct comparative data remain scarce in the literature (20, 22).

### Donor-specific characteristics influencing outcome

The literature on donor-specific allograft characteristics in vascular surgery is limited. In particular, no literature is available for comparing, or even giving any detailed information on donor-specific parameters in applications in aortic and non-aortic positions. Donor age is a recognized risk factor for allograft structural failure with older donor age as an independent predictor for structural deterioration in a large series of 2,042 aortic allograft root replacements (23). Younger donor age has been associated with reintervention after right ventricular outflow tract reconstruction, suggesting an optimal middle-aged donor window (9). With a mean age of 46 years in donors, our cohort tends to fall within this middle age range, despite the lack of a clear age definition and comparability due to confounding factors such as hemodynamic differences. Regarding donor gender, the available evidence suggests no significant impact on allograft outcomes (24). Although the aorta was acknowledged as an organ (25), several other immunogenic factors (26) contrast with solid organ transplantation, where female gender of the donor is associated with lower graft survival in male recipients (27). However, in cardiovascular medicine, it is well known that vessel quality is influenced not only by age or gender, but also by comorbidities and lifestyle factors. Therefore, from the authors' point of view, it seems more sensible to include histological quality as one of the parameters that may be decisive for prognosis, as this approach would consider the effects of possible risk factors for atherosclerosis regardless of age or gender. Histological quality of cryopreserved arterial allografts is an underreported parameter in the vascular surgery literature, with scarce available evidence derived from cardiac surgery. In aortic allograft valve implantation, graft morphology, including mineralization patterns and layered structure preservation, affects allograft durability. Grafts with moderately elevated mineralization but well-preserved layered structure and sporadic infective changes demonstrated the greatest longevity (28). The American Heart Association attributes allograft degeneration over time to prolonged storage and immune rejection. However, histological grading systems and their correlation with clinical outcomes have not been systematically evaluated for aortic or peripheral reconstructions (2). Given the paucity of data linking pre-implantation histological grading to outcomes in vascular graft infections, systematic evaluation of histological quality criteria, such as those applied in our cohort, may represent a valuable contribution toward optimizing allograft selection, though its prognostic relevance for durability remains to be established. The impact of storage duration and temperature on cryopreserved arterial allograft outcomes remains incompletely defined. Experimental studies demonstrate that the thrombogenic potential of femoral artery allografts stored at −80 °C declines during 6-month storage, though transient increased platelet adhesion occurs during the initial 3-months post-cryopreservation (29). Cryopreserved allografts show markedly lower immunogenicity compared to cold-stored allografts (stored in organ conservation solutions and transplanted within 48h). This is associated with preserved aortic wall morphology, reduced cell- and antibody-mediated immune activation (30). Touma et al. compared two cryopreservation protocols (−80 °C vs. −150 °C) and found no significant difference in early mortality or postoperative complication rates between protocols (14).

Our data demonstrate comparable outcomes to the published literature, with primary graft patency of 88% and graft patency of 78% during follow-up, alongside low rates of bleeding complications, anastomotic complications, and reinfection. These results align with reported primary patency rates of 80%–93% at 1–3 years for aortic allografts and acceptable late conduit-related complication rates of approximately 15% for peripheral reconstructions (15, 20, 31). The considerably shorter follow-up in the present cohort precludes direct comparison with these long-term data and may not allow conclusions regarding late structural degeneration. Histological graft quality may represent an interesting predictive parameter for allograft durability. However, this factor warrants further evaluation in larger studies with extended follow-up periods. It should be emphasized that this study did not demonstrate a statistically significant association between histological quality and graft patency or reintervention rates, which were comparable between groups despite differing histological grades. The observed difference in histological grading between aortic and non-aortic allografts is therefore best regarded as hypothesis-generating rather than being evidence of an independent predictive relationship with durability.

### Limitations

This study has several limitations that warrant consideration. The retrospective, single-center design inherently limits the generalizability of our findings and introduces potential selection bias. Approximately one quarter of patients underwent allograft implantation for indications other than confirmed vascular graft infection. Nonetheless, only 2 out of 51 patients (3.92%) lacked any accompanying local or systemic infectious or inflammatory findings, while the remaining 49 patients (96.08%) were operated on in an infected or inflammatory vascular field, supporting the clinical comparability of the cohort. Implantation site was chosen as the primary comparator rather than clinical indication, as position determines hemodynamic load, surgical complexity, and local infection microenvironment, whereas indications were too heterogeneous and unevenly distributed across the cohort to permit meaningful stratified comparison. The relatively small sample size (*N* = 51 patients) substantially limits statistical power and precluded multivariable adjustment to disentangle these effects. This is exemplified by the mortality comparison, where a marked absolute difference between groups (AP: 22.73% vs. NAP: 3.45%) failed to reach statistical significance (*p* = .073), underscoring the risk of type II error and precluding robust subgroup or multivariate analyses of predictive factors. Because implantation site was closely associated with operative time, blood loss, transfusion requirement and complication severity, it should be regarded as a surrogate marker of disease severity and procedural complexity rather than an independently causal determinant of survival. Another limitation is the uncertainty which of the implanted allografts caused complications. As some patients had more than one allograft implanted, it has not been possible to attribute the complications to a particular allograft. Furthermore, the median follow-up of only 193 days is likely insufficient to capture late structural or aneurysmal degeneration, delayed infection, or long-term patency loss, all of which are recognized concerns specific to cryopreserved allografts and may take years to manifest. The reported patency and reintervention rates should therefore be regarded as early/midterm outcomes only. In addition, postoperative surveillance imaging was not performed on a uniform, protocol-driven schedule across all patients, which may have led to heterogeneous detection sensitivity for subclinical graft complications and reinfection between the aortic and non-aortic groups. The lack of a control group receiving alternative conduits (e.g., autologous vein, bovine grafts, or rifampicin-bonded prostheses) preclude direct comparison of allograft performance with other treatment modalities. Finally, as with all observational studies in vascular graft infection research, unmeasured confounding variables such as variations in surgical technique, perioperative antibiotic regimens, and institutional practices may have influenced outcomes. Prospective, multicenter studies with longer follow-up periods are needed to determine whether histological graft quality independently predicts allograft durability.

## Conclusion

Cryopreserved arterial allografts constitute a valuable reconstructive option for complex vascular graft infections and selected inflammatory vascular diseases when autologous conduits are unavailable. Despite comparable graft patency and reintervention rates, patients undergoing aortic reconstruction exhibited poorer overall survival than those receiving non-aortic reconstructions. Given the close association between implantation site and operative time, blood loss and complication burden, this difference likely reflects greater disease severity and procedural complexity rather than an independent effect of the anatomical position itself. Donor-specific characteristics were largely comparable between groups. The significantly higher histological quality observed in non-aortic allografts did not correspond to differences in graft patency or reintervention rates in this cohort, but represents a hypothesis-generating finding warranting dedicated prospective evaluation. For example, further investigation is warranted to determine whether precise classification of cryopreserved grafts according to their AHA stage could provide additional value for a more comprehensive assessment of graft quality. Standardized assessment of histological graft quality may therefore complement conventional donor selection criteria and deserves further investigation in prospective multicenter studies with longer follow-up to clarify its impact on allograft durability and clinical outcomes.

## Data Availability

The raw data supporting the conclusions of this article will be made available by the authors, without undue reservation.
