# Supplementary material for: Aortic versus non-aortic cryopreserved allograft and donor-specific outcome: a retrospective observational study
Source: Front Surg. 2026 Oct 9;13:1957142. doi: 10.3389/fsurg.2026.1957142 (PMC13690762; doi:10.3389/fsurg.2026.1957142)
Supplement: Supplementary file 1 [file Datasheet1.pdf]

Supplementary Material

Supplemental Material Table S1: Histological grading criteria for allograft quality assessment according to the German Society for Tissue Transplantation (DGFG) institutional classification and corresponding American Heart Association (AHA) tissue type.

**DGFG Institutional Grade**	**Corresponding AHA Type**	**Layered Wall Architecture**	**Endothelial/ Cellular Integrity**	**Collagen Matrix**	**Mineralization/ Calcification**	**Inflammatory Infiltrate**	**Eligibility**
Grade 4 (highest quality)	Type 1–2	Fully preserved, well-defined layers	Intact endothelium, preserved cellularity	Intact, non-fragmented	None to minimal, focal	None to sparse	Eligible for implantation
Grade 3	Type 3–5	Largely preserved, mild architectural disruption	Partial endothelial loss, reduced cellularity	Largely preserved, mild fragmentation	Mild, non-confluent	Mild, focal	Eligible for implantation
Grade ≤2 (lowest quality)	Type 6	Loss of layered structure	Absent endothelium, marked acellularity	Fragmented/disorganized	Moderate to extensive, confluent	Moderate to marked	Not eligible for implantation

Supplemental Material Table S2: Distribution of preoperative and intraoperative microbiological findings by organism class in aortic versus non-aortic position allograft recipients. Organisms detected within each category are listed individually below the corresponding summary row. For polymicrobial cultures, individual isolates are denoted as gram-positive (+) or gram-negative (–).

Timing	Organism Class	Aortic Position (n = 22)	Non-aortic Position (n = 29)
Preoperative	Gram-positive only	1 (4.55 %)	3 (10.34 %)
	Gram-negative only	1 (4.55 %)	4 (13.79 %)
	Polymicrobial	0 (0.00 %)	4 (13.79 %)
	Fungal	0 (0.00 %)	0 (0.00 %)
Intraoperative Wound/Graft	Gram-positive only	3 (13.64 %)	13 (44.83 %)
	Gram-negative only	4 (18.18 %)	5 (17.24 %)
	Polymicrobial	0 (0.00 %)	1 (3.45 %)
	Fungal	0 (0.00%)	0 (0.00 %)
Preoperative	Gram-positive only	*Enterococcus faecalis*	*Enterococcus faecium, Staphylococcus haemolyticus, Staphylococcus epidermidis*

	Gram-negative only	*Pseudomonas aeruginosa*	*Escheria coli, Finegoldia magna, Pseudomonas aeruginosa, Serratia marscensens, Enterobacter cloacae*
	Polymicrobial	*none*	*Staphylococcus aureus (+), Enterococcus faecalis (+), Proteus mirabilis (-), Serratia fonticola (-), Achromobacter dentrificans (-), Citrobacter freundii (-)*
	Fungal	*none*	*none*
Intraoperative Wound/Graft	Gram-positive only	*Staphylococcus capitis, Staphylococcus aureus, Enterococcus faecalis*	*Staphylococcus epidermidis, Staphylococcus aureus, Enterococcus faecalis, Enterococcus faecium,*

	Gram-negative only	*Pseudomonas aeruginosa, Pantoea agglomerans*	*Citrobacter freundii, Escheria coli, Serratia fonticola, Achromobacter xylosoxidans, Enterobacter cloacae, Proteus mirabilis, Corynebacterium tuberculostaticum*
	Polymicrobial	*none*	*none*
	Fungal	*none*	*none*

Supplemental Material Table S3: Tabular overview of perioperative antibiotic therapy. Antimicrobial treatment was continuously optimized in collaboration with the hospital's antimicrobial stewardship team based on microbiological analyses of intraoperatively collected specimens. Arrows (→) indicate escalation or de-escalation of the antimicrobial regimen.

Patient ID	Preoperative Antimicrobial Treatment	Postoperative Antimicrobial Treatment
**1**	Clindamycin	Clindamycin → Cotrimoxazol
**2**	None	Ampicillin/Sulbactam
**3**	Meropenem + Vancomycin	Meropenem + Vancomycin → Ampicillin/Sulbactam
**4**	Ampicillin/Sulbactam	Ampicillin/Sulbactam → Cefotaxim

**5**	Clindamycin	Clindamycin → Piperacillin/Tazobactam
**6**	Piperacillin/Tazobactam + Vancomycin	Piperacillin/Tazobactam + Vancomycin → Ceftazidim + Vancomycin + Metronidazol
**7**	None	None
**8**	Clindamycin	Clindamycin → Piperacillin/Tazobactam
**9**	Cefotaxim + Metronidazol	Cefotaxim + Metronidazol → Clarithromycin + Vancomycin
**10**	Meropenem + Vancomycin	Meropenem + Vancomycin → Meropenem → Cotrimoxazol
**11**	Meropenem	Meropenem
**12**	Meropenem + Vancomycin	Meropenem + Vancomycin → Ampicillin/Sulbactam
**13**	Ceftriaxon	Ceftriaxon → Linezolid
**14**	Meropenem + Daptomycin	Meropenem + Daptomycin
**15**	Clindamycin	Clindamycin → Meropenem
**16**	Piperacillin/Tazobactam	Piperacillin/Tazobactam → Meropenem + Daptomycin + Ciprofloxacin + Caspofungin
**17**	None	Linezolid
**18**	Meropenem	Meropenem → Linezolid
**19**	Meropenem + Fosfomycin+ Daptomycin	Meropenem + Fosfomycin + Daptomycin
**20**	Meropenem + Fosfomycin	Meropenem + Fosfomycin → Ampicillin/Sulbactam

**21**	Clindamycin	Clindamycin → Piperacillin/Tazobactam → Meropenem + Fosfomycin + Daptomycin
**22**	None	None
**23**	Cefuroxim	Cefuroxim → Linezolid → Ampicillin/Sulbactam
**24**	Clindamycin	Clindamycin → Meropenem + Vancomycin
**25**	Clindamycin	Clindamycin
**26**	Clindamycin	Clindamycin
**27**	Cefazolin	Cefazolin
**28**	None	Cefuroxim + Metronidazol
**29**	None	Meropenem + Vancomycin + Daptomycin
**30**	Clindamycin	Clindamycin → Meropenem + Linezolid + Vancomycin → Linezolid
**31**	None	Cefotaxim → Meropenem + Fosfomycin + Daptomycin + Caspofungin
**32**	Ampicillin/Sulbactam	Ampicillin/Sulbactam → Meropenem
**33**	Ampicillin/Sulbactam	Ampicillin/Sulbactam
**34**	Ceftazidim	Ceftazidim → Meropenem
**35**	None	Ampicillin/Sulbactam

**36**	None	Piperacillin/Tazobactam
**37**	Meropenem + Fosfomycin	Meropenem + Fosfomycin + Daptomycin → Linezolid
**38**	Meropenem + Fosfomycin	Meropenem + Fosfomycin → Cefazolin → Rifampicin + Doxycyclin
**39**	Meropenem	Meropenem + Fosfomycin + Daptomycin
**40**	Meropenem	Meropenem → Ceftriaxon
**41**	Ampicillin/Sulbactam	Ampicillin/Sulbactam
**42**	Ampicillin/Sulbactam	Ampicillin/Sulbactam → Meropenem + Daptomycin
**43**	Ampicillin/Sulbactam	Ampicillin/Sulbactam → Meropenem
**44**	None	Meropenem + Daptomycin
**45**	None	Meropenem → Linezolid
**46**	Meropenem + Daptomycin	Meropenem + Daptomycin → Ciprofloxacin
**47**	Amoxicillin/Clavulanate	Amoxicillin/Clavulanate → Meropenem + Vancomycin
**48**	Ampicillin/Sulbactam	Ampicillin/Sulbactam
**49**	None	None
**50**	None	None
**51**	Meropenem	Meropenem
